# Cardiovascular Indicators of Systemic Circulation and Acute Mountain Sickness: An Observational Cohort Study

**DOI:** 10.3389/fphys.2021.708862

**Published:** 2021-08-27

**Authors:** Renzheng Chen, Mengjia Sun, Jie Yang, Chuan Liu, Jihang Zhang, Jingbin Ke, Yuhan Deng, Chunyan He, Yuanqi Yang, Ran Cheng, Fangzhengyuan Yuan, Hu Tan, Xubin Gao, Lan Huang

**Affiliations:** ^1^Institute of Cardiovascular Diseases of Chinese People's Liberation Army, The Second Affiliated Hospital, Third Military Medical University (Army Medical University), Chongqing, China; ^2^Department of Cardiology, The Second Affiliated Hospital, Third Military Medical University (Army Medical University), Chongqing, China

**Keywords:** ambulatory blood pressure, cardiac ultrasound, cardiovascular indicators of systemic circulation, acute mountain sickness, high altitude

## Abstract

**Background:** Acute high-altitude (HA) exposure results in blood pressure (BP) and cardiac function variations in most subjects, some of whom suffer from acute mountain sickness (AMS). Several previous studies have found that cardiovascular function indicators are potentially correlated with AMS.

**Objectives:** This study aims to examine HA-induced cardiovascular adaptations in AMS patients and compare them with healthy subjects. It also aims to investigate the relationship between cardiovascular function indicators and AMS, as well as to provide some insightful information about the prevention and treatment of AMS.

**Methods:** Seventy-two subjects were enrolled in this cohort study. All the subjects ascended Litang (4,100 m above sea level). They were monitored by a 24-h ambulatory blood pressure (ABP) device and underwent echocardiography examination within 24 h of altitude exposure. The 2018 Lake Louise questionnaire was used to evaluate AMS.

**Results:** Acute mountain sickness group consisted of more women (17 [60.7%] vs. 10 [22.7%], *p* = 0.001) and fewer smokers (5 [17.9%] vs. 23 [52.3%], *p* = 0.003). Compared with subjects without AMS, subjects with AMS had lower pulse pressure (PP) (daytime PP, 45.23 ± 7.88 vs. 52.14 ± 4.75, *p* < 0.001; nighttime PP, 42.81 ± 5.92 vs. 49.39 ± 7.67, *p* < 0.001) and lower effective arterial elastance (Ea) (1.53 ± 0.24 vs. 1.73 ± 0.39, *p* = 0.023). Multivariate regression indicated that female sex (OR = 0.23, *p* = 0.024), lower daytime PP (OR = 0.86, *p* = 0.004), and lower Ea (OR = 0.03, *p* = 0.015) at low altitude (LA) were independent risk factors for AMS. Combined daytime PP and Ea at LA had a high predictive value for AMS (AUC = 0.873; 95% CI: 0.789–0.956). Correlation analysis showed that AMS-induced headache correlated with daytime PP (*R* = −0.401, *p* < 0.001) and nighttime PP at LA (*R* = −0.401, *p* < 0.001).

**Conclusion:** Our study demonstrated that AMS patients had a lower PP and Ea at LA. These baseline indicators of vasodilation at LA were closely associated with AMS, which may explain the higher headache severity in subjects with higher PP at LA.

## Introduction

An increasing number of people have access to high-altitude (HA) areas for various reasons. However, HA is associated with hypobaric and hypoxic conditions and can induce a series of cardiovascular adaptations (Hainsworth and Drinkhill, 2007; Naeije, 2010). Some healthy individuals with poor cardiac acclimation may suffer from acute mountain sickness (AMS), which is characterized, among other symptoms, by dizziness, headache, nausea, insomnia, vomiting, and fatigue (Luks et al., 2017). Independent risk factors for AMS, such as genetics, sex, body mass index (BMI), and the ascending altitude and velocity, have been confirmed in previous studies (Santantonio et al., 2014; Luks et al., 2017). Moreover, previous findings have also demonstrated that AMS may be affected by the baseline and HA-induced changes of cardiovascular indices (Lanfranchi et al., 2005; Hamm et al., 2020; Ke et al., 2020). However, conflicting results are often reported and the exact mechanism of AMS still needs to be fully elucidated.

Two parameters, pulse pressure (PP) and arterial elastance (Ea), which can be easily measured noninvasively, are important for determining the systolic and diastolic compliance of blood vessels. It has been previously shown that higher PP is a risk factor for cardiovascular and cerebrovascular events, such as ischemic stroke (Liu et al., 2016). PP is a consequence of the episodic nature of cardiac contraction and the properties of the arterial circulation (Dart and Kingwell, 2001). Ea, which is defined as the ratio of end-systolic left ventricular (LV) pressure and stroke volume, increases with increasing vascular stiffness (Ikonomidis et al., 2019). However, few studies have focused on the vascular systolic and diastolic function during HA exposure, especially in subjects with AMS and less information exists about the relationship between these functional indices and AMS.

Thus, in the present study, we collected data on 24-h ambulatory blood pressure (ABP) and echocardiography at both low altitude (LA) and HA in AMS patients and non-AMS controls. We sought to determine whether there was a relationship between differential cardiovascular adaptation in HA and AMS. Moreover, we sought to provide some insights on the prevention and treatment of AMS.

## Materials and Methods

### Study Population and Ethical Considerations

Ninety-one subjects (32 women and 59 men) were included in this prospective observational cohort study that was performed in Chengdu, China, in 2019. Informed consent was obtained, and all subjects underwent a comprehensive medical examination before the expedition to LA (Chongzhou, 400 m). Information on cigarette smoking and alcohol history was collected from all participants. Subjects underwent ABP monitoring and echocardiography both at LA and HA (Litang, 4,100 m above sea level). Exclusions were listed as follows: (1) Any clinical conditions that may affect HA-adaptation, including known pulmonary diseases, cardiovascular diseases, hematological diseases, (2) use of any oral medication. Ten subjects were excluded due to the above and another nine subjects were excluded due to lack of data. In total, 72 subjects were included, all of whom were volunteers who signed written informed consent. This study was conducted in agreement with the Declaration of Helsinki and was approved by the Human Ethics Committee of the Xinqiao Hospital, Third Military Medical University (Identification code, 201907501) which was registered at www.chictr.org.cn (ChiCTR-TRC-No.1900025728).

### BP Measurement and BP Parameters Calculation

Two well-trained cardiovascular physicians recorded BP data using an ABP measurement device (Spacelabs 90207, Redmond, WA, USA). The BP cuff was applied to the nondominant arm on a weekday morning and was removed 24 h later. All participants were asked to remain still during the measurement. The subjects were instructed to avoid unusual physical activities, and follow a standard schedule at both locations, LA and HA. Daytime and nighttime were defined as 6:00 to 22:00 and 22:00 to 6:00, respectively (Georgianos and Agarwal, 2015). The recorders measured BP every 30 min during daytime and every 60 min during nighttime (Huang et al., 2019). PP was the difference between systolic blood pressure (SBP) and diastolic blood pressure (DBP). The nocturnal decline in BP was recorded and the average real variability (ARV) of SBP and DBP was calculated as described in previous study (Mena et al., 2005). ARVs and ARVd represent the ARV of SBP and DBP, respectively.

### Calculation of the Left Ventricular Function Parameters

Echocardiographic examination was performed using an ultrasound machine (CX50, Philips Ultrasound System, Andover, MA, USA) to acquire LV data. Images were saved digitally for subsequent offline analysis using QLAB software (QLAB 10.5, Philips Healthcare, Andover, MA, USA). Measurements of LV dimensions and volumes were performed by a computerized analysis software system. Ejection fraction (EF) was calculated by the LV volume data. Mitral inflow pattern from the tips level was analyzed for peak early diastolic velocity (E) as well as late diastolic velocity (A) and E/A. Mitral annulus early diastolic velocity (e′) was measured at the septal and lateral mitral annulus and the E/e′ ratio was calculated by the mean septal E/e′ ratio and mean lateral E/e′ ratio. Effective Ea and end-systolic elastance (Ees) were computed by the formulas: SBP × 0.9/stroke volume and SBP × 0.9/end-systolic volume (ESV), whereas ventricular-arterial decoupling (VAC) was the ratio of Ea and Ees (Ikonomidis et al., 2019). We used a two-dimensional ultrasound speckle tracking imaging technique to measure LV torsion, LV untwisting rate, and LV strain. The software automatically selects suitable stable objects for tracking and then searches for them in the next frame using a sum of absolute differences algorithm. We defined LV torsion as the difference between the apical and basal angle during systole around the longitudinal LV axis relative to the starting position, and the untwisting rate was the maximum untwisting velocity calculated by the angle during diastole (Gnakamene et al., 2018). The global longitudinal strain (GLS) was calculated by averaging all the values of the regional peak longitudinal strain obtained in the two-, three-, and four-chambered apical views. The global circumferential strain (GCS) was assessed as the average of three LV regional values measured in the parasternal short-axial view at the basal level. Measurements of three cardiac cycles were averaged.

### Assessment of AMS

All the subjects ascended from LA to HA within 2 days. The latest Lake Louise questionnaire (2018) was used to assess AMS 8 h after arriving at HA. Participants completed a four-item questionnaire with the assistance of an experienced physician. The questions involved the presence of headache, dizziness or lightheadedness, gastrointestinal symptoms, and fatigue. Each question had four possible responses ranging from 0 to 3, according to the severity of the symptoms (0 for no symptoms, 1 for mild symptoms, 2 for moderate symptoms, and 3 for severe symptoms). AMS was defined as a total score ≥3 with at least 1 point from headache (Roach et al., 2018).

### Echocardiography Reproducibility

Reproducibility of main echocardiographic measurements was assessed in 20 randomly selected subjects. Interobserver variability was tested by two different physicians, and intraobserver variability was tested by the same physician at least 1 month apart. Both the interobserver and intraobserver variabilities were determined using the intraclass correlation coefficient (ICC). Corresponding results were listed in the Supplementary Table 2.

### Statistical Analysis

Continuous variables were presented as mean ± standard deviation. Differences in measurements between men and women with normal distribution were tested using an independent sample *t*-test, while the data that did not fit a normal distribution were analyzed by the Mann–Whitney *U*-test. Categorical data were presented as percentages (%) and were compared by the chi-square test, continuity correction, or Fisher's exact test, as appropriate. Binary and multivariate logistic regression was used to predict the risk factors of AMS, and receiver operating characteristic (ROC) curve was computed to evaluate the effectiveness of the prediction. Given the collinearity between variables and the number of subjects available, variables for inclusion were carefully chosen in our model. In addition, due to headache being the core symptom of AMS, we used a multiple linear regression model to evaluate the relationship between the risk factors of AMS and HA-related headache. Statistical significance was assumed at *p* < 0.05. Statistical analyses were performed by using SPSS software 26 (IBM, Armonk, NY, USA).

## Results

### Basic and AMS-Related Symptoms Parameters

Twenty-eight subjects developed AMS in the final data analysis. Age and BMI did not differ statistically between the two groups. The AMS group had a higher proportion of women (60.7 vs. 22.7%, *p* = 0.001) but a lower rate of smokers (17.9 vs. 52.3%, *p* = 0.003). The score of AMS and the percentage of AMS-related symptoms were also significantly higher in the AMS group (Table 1).

**Table 1:** Demographic and AMS symptoms parameters.

**Variables**	**All (*n* = 72)**	**AMS (*n* = 28)**	**Non-AMS (*n* = 44)**	***P*-value**
Age, years	26.99 ± 7.87	28.07 ± 8.95	26.30 ± 7.12	0.354
BMI, kg/m^2^	21.82 ± 2.17	21.64 ± 2.24	21.93 ± 2.14	0.577
Females	27 (37.5%)	17 (60.7%)	10 (22.7%)	0.001
Tibetan	1 (1.4%)	0 (0.0%)	1 (2.3%)	1.000
Alcohol	11 (15.3%)	6 (21.4%)	5 (11.4%)	0.247
Cigarette smoking	28 (38.9%)	5 (17.9%)	23 (52.3%)	0.003
AMS score	2.50 ± 1.86	4.39 ± 1.42	1.30 ± 0.79	<0.001
Headache	51 (70.8%)	28 (100.0%)	23 (52.3%)	<0.001
Dizziness	39 (54.2%)	24 (85.7%)	15 (34.1%)	<0.001
Gastrointestinal symptoms	13 (18.1%)	11 (39.3%)	2 (4.5%)	<0.001
Fatigue	43 (59.7%)	28 (100.0%)	15 (34.1%)	<0.001

*Values are presented as mean ± standard deviation and percentage (%)*.

*BMI, body mass index; AMS, acute mountain sickness*.

### Effects of Acute HA Exposure on BP and LV Cardiac Function

Daytime SBP, nighttime SBP, daytime DBP, and nighttime DBP increased after acute exposure to HA in all subjects. Heart rate (HR) also significantly increased, whereas SpO_2_ decreased (Supplementary Table 1). No statistical difference was found in SpO_2_ between the two groups at both LA and at HA. Patients with AMS had a higher nighttime HR (62.33 ± 8.18 vs. 58.43 ± 9.66, *p* = 0.018) and a lower daytime SBP (118.23 ± 13.23 vs. 124.38 ± 5.39, *p* = 0.007) at LA. Interestingly, although DBP was similar, daytime and nighttime PP were significantly lower in AMS subjects when compared with non-AMS subjects at LA (daytime PP 45.23 ± 7.88 vs. 52.14 ± 4.75, *p* < 0.001; nighttime PP 42.81 ± 5.92 vs. 49.39 ± 7.67, *p* < 0.001). After arriving at HA, PP increased in the AMS group but declined in the non-AMS group. Furthermore, there was no obvious difference in ARV between the two groups both at LA and at HA (Table 2).

**Table 2:** Effect of acute HA exposure.

**Variables**	**LA**			**HA**			**Delta LA-HA**		
	**AMS (*n* = 28)**	**Non-AMS (*n* = 44)**	***P*-value**	**AMS (*n* = 28)**	**Non-AMS (*n* = 44)**	***P*-value**	**AMS (*n* = 28)**	**Non-AMS (*n* = 44)**	***P*-value**
SpO_2_, %	97 ± 2	97 ± 1	0.076	87 ± 3	87 ± 3	0.726	−10 ± 4	−10 ± 4	0.625
Daytime HR, bpm	78.21 ± 8.61	76.59 ± 7.52	0.402	88.69 ± 6.94	87.73 ± 7.98	0.603	10.48 ± 8.61	11.14 ± 7.87	0.738
Nighttime HR, bpm	62.33 ± 8.18	58.43 ± 9.66	0.018	74.37 ± 11.64	68.75 ± 9.66	0.029	12.04 ± 9.67	10.32 ± 10.83	0.496
**BP characteristic, mmHg**									
Nocturnal SBP fall, %	9.77 ± 9.56	11.52 ± 8.66	0.425	10.81 ± 8.53	12.23 ± 7.21	0.451	1.04 ± 12.20	0.71 ± 9.94	0.900
Nocturnal DBP fall, %	13.36 ± 9.09	15.88 ± 9.54	0.270	13.73 ± 9.55	14.94 ± 10.09	0.616	0.37 ± 12.40	−0.94 ± 12.92	0.671
Daytime SBP	118.23 ± 13.23	124.38 ± 5.39	0.007	126.68 ± 9.73	130.81 ± 9.33	0.076	8.45 ± 10.40	6.44 ± 9.10	0.390
Nighttime SBP	105.93 ± 10.66	109.89 ± 10.31	0.121	112.96 ± 13.96	114.58 ± 9.99	0.568	7.03 ± 9.19	4.69 ± 11.53	0.368
Daytime DBP	73.01 ± 9.14	72.14 ± 4.45	0.594	78.27 ± 6.34	78.43 ± 5.18	0.905	5.26 ± 8.11	6.29 ± 5.31	0.556
Nighttime DBP	63.13 ± 9.93	60.51 ± 6.31	0.175	67.63 ± 10.22	66.59 ± 7.99	0.629	4.51 ± 8.76	6.08 ± 8.11	0.439
Daytime PP	45.23 ± 7.88	52.14 ± 4.75	<0.001	48.09 ± 7.31	52.19 ± 7.81	0.029	2.86 ± 8.17	0.05 ± 8.58	0.172
Nighttime PP	42.81 ± 5.92	49.39 ± 7.67	<0.001	45.33 ± 7.68	47.85 ± 7.13	0.160	2.52 ± 8.18	−1.54 ± 7.94	0.040
Daytime ARVs	17.45 ± 6.06	18.49 ± 5.05	0.431	21.08 ± 6.96	21.33 ± 5.17	0.861	3.63 ± 7.36	2.84 ± 5.84	0.614
Nighttime ARVs	12.78 ± 6.02	14.60 ± 7.23	0.272	14.16 ± 6.70	12.84 ± 6.05	0.389	1.38 ± 8.44	−1.76 ± 10.34	0.183
Daytime ARVd	13.80 ± 5.75	14.20 ± 4.29	0.733	16.22 ± 5.87	17.42 ± 11.45	0.608	2.42 ± 8.38	1.74 ± 6.44	0.700
Nighttime ARVd	9.14 ± 4.53	11.04 ± 7.51	0.232	10.56 ± 4.02	10.87 ± 5.41	0.794	1.41 ± 6.13	−0.17 ± 9.33	0.431
**Cardiac function characteristic**									
EDV, ml	115.37 ± 19.38	108.76 ± 22.81	0.209	105.44 ± 20.41	100.60 ± 17.72	0.291	−12.90 ± 19.69	−6.26 ± 22.98	0.211
ESV, ml	47.87 ± 7.84	44.03 ± 11.64	0.129	42.10 ± 11.67	39.29 ± 9.80	0.275	−6.04 ± 12.22	−4.57 ± 12.83	0.631
EF, %	58.40 ± 3.66	59.91 ± 4.49	0.141	60.30 ± 6.59	61.16 ± 5.62	0.555	0.98 ± 7.07	1.83 ± 5.83	0.582
E/A	1.84 ± 0.63	1.89 ± 0.56	0.707	1.44 ± 0.35	1.42 ± 0.35	0.786	−0.39 ± 0.57	−0.47 ± 0.59	0.603
E/e'	6.58 ± 1.04	6.57 ± 1.10	0.733	5.93 ± 1.26	5.78 ± 1.20	0.625	−0.65 ± 1.20	−0.79 ± 1.17	0.638
GLS, %	19.98 ± 1.98	20.34 ± 2.06	0.461	20.46 ± 2.67	21.25 ± 2.49	0.206	0.48 ± 2.21	0.91 ± 1.99	0.396
GCS, %	25.29 ± 2.26	25.87 ± 2.94	0.112	25.14 ± 2.41	25.18 ± 2.46	0.945	−0.15 ± 2.85	−0.70 ± 3.13	0.459
Untwisting rate, °/s	74.88 ± 42.34	79.01 ± 30.03	0.630	95.66 ± 47.52	99.67 ± 33.68	0.676	20.78 ± 30.46	20.67 ± 27.71	0.987
Torsion, °	10.95 ± 3.30	11.95 ± 3.45	0.228	13.98 ± 4.55	14.56 ± 4.62	0.602	2.99 ± 3.78	2.62 ± 5.08	0.742
Ees, mmHg/ml	2.16 ± 0.39	2.69 ± 1.21	0.005	2.79 ± 0.86	2.98 ± 0.88	0.376	0.62 ± 0.96	0.29 ± 1.41	0.283
Ea, mmHg/ml	1.53 ± 0.24	1.73 ± 0.39	0.023	1.78 ± 0.35	1.85 ± 0.41	0.463	0.31 ± 0.38	0.08 ± 0.53	0.052
VAC	0.72 ± 0.11	0.68 ± 0.12	0.138	0.68 ± 0.20	0.65 ± 0.16	0.488	−0.01 ± 0.20	−0.05 ± 0.17	0.425

*Values are presented as mean ± standard deviation*.

*HA, high altitude; LA, low altitude; AMS, acute mountain sickness; HR, heart rate; BP, blood pressure; SBP, systolic blood pressure; DBP, diastolic blood pressure; PP, pulse pressure; ARVs, average real variability of SBP; ARVd, average real variability of DBP; GLS, global longitudinal strain; GCS, global circumferential strain; EDV, end-diastolic volume; ESV, end-systolic volume; Ees, end-systolic elastance; Ea, effective arterial elastance; VAC, ventricular-arterial coupling; EF, ejection fraction; E/A, peak early diastolic velocity/late diastolic velocity; E/e′, peak early diastolic velocity/early diastolic velocity*.

End-diastolic volume (EDV) and ESV decreased significantly in all subjects after HA exposure. E/A and E/e′ declined but LV torsion and untwisting rate increased. This indicated that diastolic relaxation was not acutely impaired. Both Ees and Ea increased in the whole cohort after arriving at HA (Supplementary Table 1). There was no difference in cardiac systolic and diastolic function between the two groups at both LA and HA. Moreover, we found that the LV cardiac mechanical indices were not significantly different. It was worth noting that, compared with non-AMS subjects, AMS patients showed a significantly lower Ees (2.16 ± 0.39 vs. 2.69 ± 1.21, *p* = 0.005) and Ea (1.53 ± 0.24 vs. 1.73 ± 0.39, *p* = 0.023) while the difference of VAC was not obvious between the two groups at LA. Ea varied more in the AMS group after HA exposure but the difference did not reach statistical significance (Table 2).

### Risk Factors for AMS

**Figure 1:**
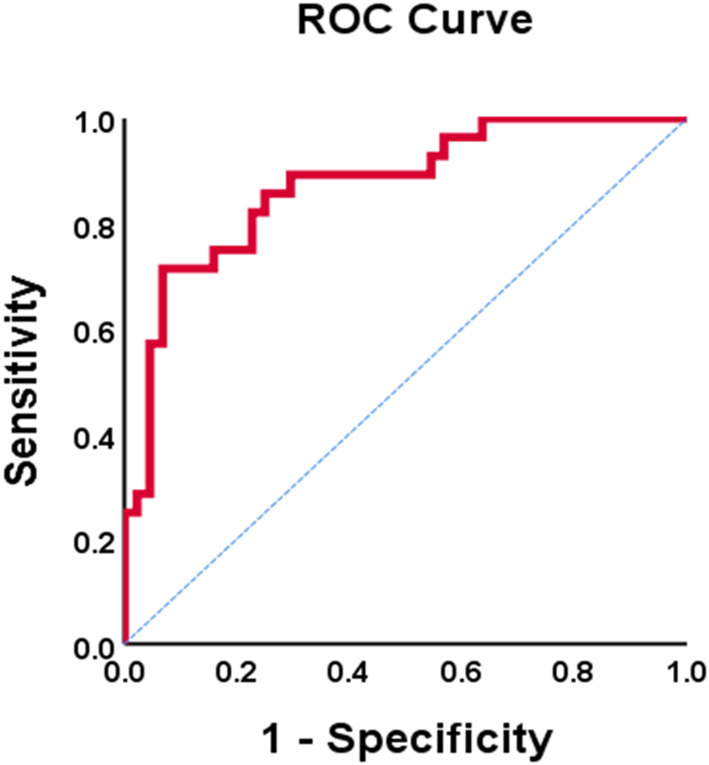
ROC curve of PP combined Ea diagnosis AMS. AMS, acute mountain sickness; PP, pulse pressure; Ea, effective arterial elastance.

In the univariate analysis that included all subjects, higher EDV (OR = 1.03, *p* = 0.035) was associated with increased risk of AMS. Smoking (OR = 0.20, *p* = 0.005), male sex (OR = 0.19, *p* = 0.002), higher daytime SBP (OR = 0.93, *p* = 0.013), higher daytime PP (OR = 0.83, *p* < 0.001), higher nighttime PP (OR = 0.86, *p* = 0.001), higher Ees (OR = 0.28, *p* = 0.020), and higher Ea (OR = 0.95, *p* = 0.001) were all associated with decreased incidence of AMS (Table 3). Multivariate regression that included all subjects identified the following risk factors for AMS: female sex (OR = 0.23, *p* = 0.024), lower daytime PP (OR = 0.86, *p* = 0.004), and lower Ea (OR = 0.03, *p* = 0.015) (Table 3). ROC curve analysis was performed to test the reliability of these risks for diagnosing AMS. Female sex [area under the curve (AUC) = 0.690; 95% CI: 0.560–0.819], lower daytime PP (AUC = 0.788; 95% CI: 0.665–0.910), and lower Ea (AUC = 0.752; 95% CI: 0.640–0.863) had good prognostic value. Combining the above parameters had a high predictive value for AMS diagnosis (AUC = 0.873; 95% CI: 0.789–0.956) (Figure 1).

**Table 3:** Risk factors of AMS.

**Variable**	**Univariable**	***P*-value**	**Multivariable**	***P*-value**
	**OR (95% CI)**		**OR (95% CI)**	
Age	1.03 (0.97–1.09)	0.356		
BMI	0.94 (0.75–1.17)	0.572		
Gender (male vs. female)	0.19 (0.07–0.54)	0.002	0.23 (0.07–0.82)	0.024
Cigarette smoking	0.20 (0.06–0.62)	0.005		
Daytime SBP	0.93 (0.88–0.99)	0.013		
Nighttime SBP	0.96 (0.92–1.01)	0.123		
Daytime DBP	1.02 (0.95–1.10)	0.589		
Nighttime DBP	1.04 (0.98–1.11)	0.182		
Daytime PP	0.83 (0.75–0.92)	<0.001	0.86 (0.78–0.95)	0.004
Nighttime PP	0.86 (0.78–0.94)	0.001		
Daytime HR	1.03 (0.97–1.09)	0.397		
Nighttime HR	1.05 (0.99–1.11)	0.087		
SpO_2_	0.73 (0.53–1.01)	0.061		
EDV	1.03 (1.00–1.05)	0.035		
ESV	1.04 (0.99–1.10)	0.095		
EF	1.00 (0.89–1.12)	0.986		
Ees	0.28 (0.10–0.82)	0.020		
Ea	0.02 (0.00–0.21)	0.001	0.03 (0.00–0.50)	0.015
E/A	0.86 (0.38–1.97)	0.726		
E/e′	1.01 (0.65–1.58)	0.970		
GLS	0.91 (0.72–1.16)	0.455		
GCS	0.92 (0.76–1.11)	0.370		
Untwisting rate	1.00 (0.99–1.02)	0.625		
Torsion	0.91 (0.78–1.06)	0.230		

*AMS, acute mountain sickness; BMI, body mass index; HR, heart rate; SBP, systolic blood pressure; DBP, diastolic blood pressure; PP, pulse pressure; GLS, global longitudinal strain; GCS, global circumferential strain; EDV, end-diastolic volume; ESV, end-systolic volume; Ees, end-systolic elastance; Ea, effective arterial elastance; EF, ejection fraction; E/A, peak early diastolic velocity/late diastolic velocity; E/e′, peak early diastolic velocity/early diastolic velocity*.

### Correlation Between Risk Factors and HA-Related Headache

**Figure 2:**
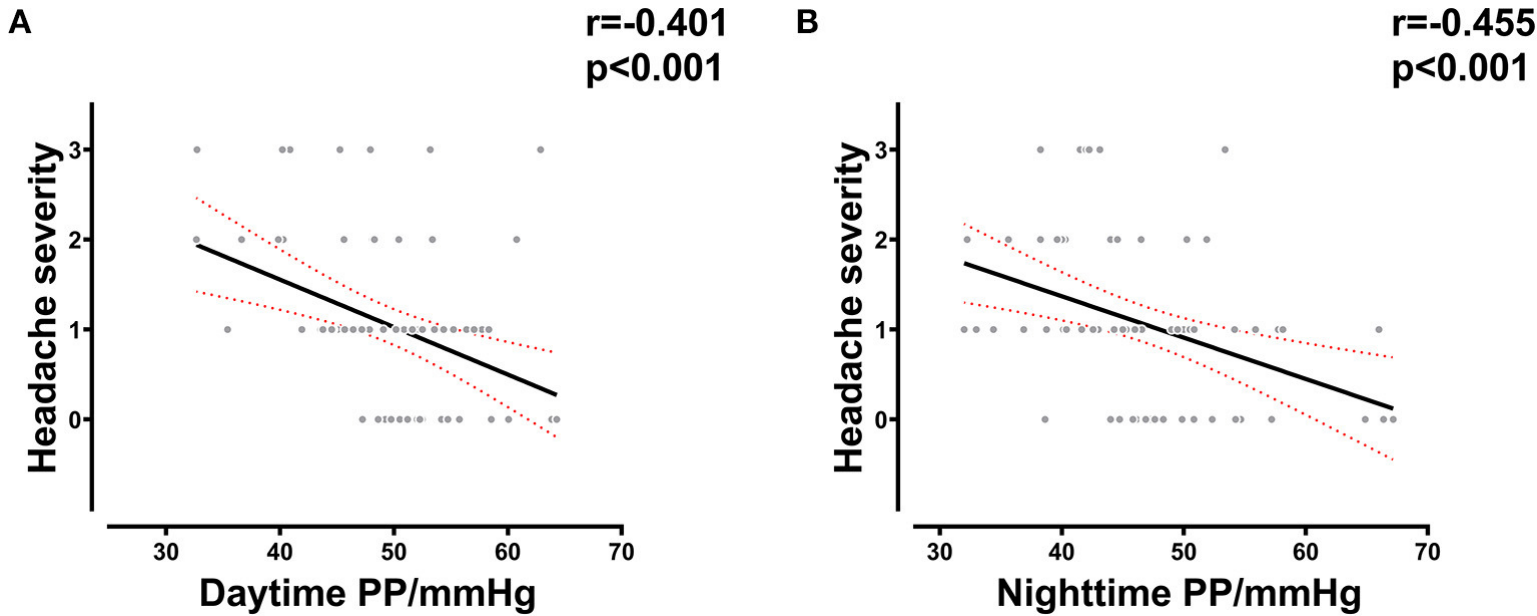
Correlation between PP and AMS-related headache severity. **(A)** Correlation between daytime PP at LA and headache severity in all subjects. **(B)** Correlation between nighttime PP at LA and headache severity in all subjects. LA, low altitude; AMS, acute mountain sickness; PP, pulse pressure.

The results of the analysis showed that the severity of headache was correlated with daytime PP (*R* = −0.401, *p* < 0.001) and nighttime PP at LA (*R* = −0.401, *p* < 0.001) (Figure 2), which indicated a correlation between vascular compliance at LA and HA-related headache. This could partly explain the association between vascular function and AMS.

## Discussion

Our study demonstrated that variations of ABP and cardiac function occur on acute HA exposure in both healthy and AMS subjects and analyzed the correlation between cardiovascular indicators and AMS. Interestingly, we found that the baseline levels of PP and Ea at LA, which represent vascular compliance, have good predictive value for AMS. This might explain the significant association between these factors and HA-induced headache.

### Cardiac Response After HA Exposure in AMS Subjects

AMS is diagnosed by nonspecific symptoms that occur a few hours after exposure to altitudes of ≥2,500 m in unacclimatized subjects (Richalet et al., 2013). People usually experience various degrees of AMS on ascending to a HA location. The rate of AMS varies from 10 to 70% in different studies (Ren et al., 2010; Luks et al., 2017). However, the characteristics of LV function-related responses in AMS patients have not been illustrated so far. Our previous reports have documented the cardiac adaptations in patients with AMS compared with healthy subjects (Ke et al., 2020). AMS patients had higher HR but lower EDV, stroke volume, and E/A ratio than AMS-free subjects. These results indicated an abnormal LV diastolic function and altered LV filling pattern due to reduced preload induced by pulmonary vasoconstriction. Additionally, a rapid decrease in high-energy phosphate metabolism during acute HA exposure may have contributed to this phenomenon (Holloway et al., 2011). However, there was no significant difference in systemic BP. Although subendocardial systolic dysfunction may have occurred, global LV function stayed normal (Osculati et al., 2016). The controversy in the results may have been due to different study contexts, environmental conditions, and study populations. In the current study, we found that nighttime HR was higher in the AMS group. We also noticed that daytime PP was lower in AMS subjects than in non-AMS individuals, which is a novel observation. These indicators may be correlated with AMS.

### Potential Risk Factors for AMS

Previous reports identified risk factors associated with AMS (Fiore et al., 2010; Santantonio et al., 2014). Women are more susceptible to AMS than men (Hou et al., 2019). This was consistent with our findings. Previous studies have also shown that alcohol use and obesity are associated with AMS (San Martin et al., 2017; Yang et al., 2020), whereas we found no difference in BMI and alcohol history between the two groups. Notably, a meta-analysis showed smoking to be neither a risk nor a protective factor, whereas we found that smoking was protective against AMS. Thus, further research might be needed on this issue (Vinnikov et al., 2016). Few studies have focused on the relationship between baseline cardiovascular indices and AMS. The autonomous nervous system may respond differently in AMS patients and healthy subjects during exposure to HA (Bourdillon et al., 2018). Transient autonomic dysfunction could result in more pronounced BP changes during initial hypoxic exposure. Differential cardiovascular adaptation caused by different sensitivity to hypoxia may be an important predisposing factor for AMS (Niebauer et al., 2020; Chen et al., 2021). Furthermore, the reduced arterial oxygen saturation has been proposed as a risk factor of AMS (Karinen et al., 2010). Notably, significant SpO_2_ variation during HA exposure was not found between the two groups in our study. The discrepancy between different studies may be due to differences in exposure time, ascent velocity, and study subjects (Guo et al., 2014). We believed that vascular function itself may be involved in the development of AMS. The baseline cardiovascular function may also be used for the prediction of AMS. In our study, we found that PP and Ea, which represent the compliance of blood vessels, were potential predictors of AMS. Combining the two indices could effectively predict AMS.

### Association Between Baseline Vascular Function and AMS

Previous studies have confirmed that high PP is an independent risk factor for adverse cardiovascular events (Liu et al., 2016). Ea represents the degree of arterial stiffness which is also associated with cardiovascular complications (Ikonomidis et al., 2019). However, few studies have investigated the correlation between vascular function and AMS. In our investigation, we revealed that baseline vascular function indices correlated with AMS. However, there was no difference in PP and Ea variation after HA exposure between the two groups. To elucidate the potential mechanism, we further analyzed the relationship between these parameters and HA-induced headache (core symptom of AMS). We found that the severity of headache was significantly associated with PP at LA. Several previous studies have shown that increased PP is related to arterial stiffness and it may decrease headache prevalence through modulation of the baroreflex arch, which generates hypoalgesia (Tronvik et al., 2008, 2011). Moreover, we also found more cigarette smokers, who always accompanied with a poorer vascular function, among non-AMS subjects compared with AMS patients. Sympathetic activation is induced by hypobaric hypoxic stimulus, and subjects with higher vascular reactivity may undergo more pronounced vascular constriction after HA exposure, thus causing a headache. Although previous small sample studies indicated that vascular endothelial dysfunction exists in AMS patients compared with no-AMS subjects during acute HA exposure (Lanfranchi et al., 2005; He et al., 2016), the exact relationship between vascular function at baseline and AMS is unknown. Our findings suggest that subjects with higher vascular reactivity are more vulnerable to hypoxic-induced altitude headache, which is associated with a higher rate of AMS. Certainly, it should be pointed out that this conclusion may be applicable within a certain PP range or even in a specific population.

### Prevention of AMS

Our findings could shed some light on the prevention of AMS. We used simple and noninvasive indicators of cardiovascular function to predict the incidence of AMS and the prediction value was significant. For subjects with some symptoms but not fully developed AMS, if baseline PP and Ea are relatively low, attention should be given and appropriate treatment should be initiated to prevent the progress toward AMS. Furthermore, our findings could be very useful in revealing the potential mechanism of AMS if they can be verified by larger cohort studies in different populations and conditions.

### Limitations

There are several limitations in our present study. First, the enrolled participants were mostly young Chinese people from the Han population. Whether the established results could extend to other ethnicities or circumstances is still unknown. Second, we did not examine parameters related to the biochemical and cerebral blood flow due to the limitations of HA conditions in this study. The potential mechanism remains to be further studied to explain this phenomenon. Finally, the diagnosis of AMS was based on a self-report, which might lead to classification bias. Also, several subjects who develop AMS at HA without the first 12 h after arrival may be classified mistakenly as non-AMS patients.

## Conclusion

So far, less information has been known about the relationship between cardiovascular indicators of systemic circulation and AMS. Our study demonstrated that the baseline level of vasodilation function indicators at LA is closely associated with AMS, which may attribute to higher headache severity in subjects with lower PP levels at LA. These results may provide novel insights into the underlying mechanisms in the occurrence of AMS and new strategies for the prevention and treatment of AMS.

## Data Availability Statement

The raw data supporting the conclusions of this article will be made available by the authors, without undue reservation.

## Ethics Statement

This study was conducted in agreement with the Declaration of Helsinki and was approved by the Human Ethics Committee of the Xinqiao Hospital, Third Military Medical University (Indentificaton code, 201907501) which registered at www.chictr.org.cn (ChiCTR-TRC-No.1900025728). The patients/participants provided their written informed consent to participate in this study. Written informed consent was obtained from the individual(s) for the publication of any potentially identifiable images or data included in this article.

## Author Contributions

ReC, JY, and LH worked on the conception of the study. ReC, MS, YY, JK, FY, CH, RaC, and LH contributed to the data collection. CL and JY checked the data. ReC and MS performed the statistical analysis. JY and ReC drafted the manuscript. YD, XG, CL, HT, JZ, and LH reviewed the manuscript. All authors have read and approved the final version of the manuscript.

## Conflict of Interest

The authors declare that the research was conducted in the absence of any commercial or financial relationships that could be construed as a potential conflict of interest.

## Publisher's Note

All claims expressed in this article are solely those of the authors and do not necessarily represent those of their affiliated organizations, or those of the publisher, the editors and the reviewers. Any product that may be evaluated in this article, or claim that may be made by its manufacturer, is not guaranteed or endorsed by the publisher.
