# Supplementary material for: Cardiovascular Indicators of Systemic Circulation and Acute Mountain Sickness: An Observational Cohort Study
Source: Front Physiol. 2021 Aug 27;12:708862. doi: 10.3389/fphys.2021.708862 (PMC8430240; doi:10.3389/fphys.2021.708862)
Supplement: Supplementary file 1 [file Data_Sheet_1.doc]

**Supplement table 1. Effect of acute HA exposure on BP in the total population.**

| **Variables** | **LA** | **HA** | **HA vs. LA** |
| --- | --- | --- | --- |
|  | **Total (n=72)** | **Total (n=72)** | **P value** |
| SpO_2_,% | 97±2 | 87±3 | <0.001 |
| Daytime HR, bpm | 77.22±7.94 | 88.10±7.55 | <0.001 |
| Nighttime HR, bpm | 59.95±9.26 | 70.94±10.76 | <0.001 |
| **BP characteristic, mmHg** |  |  |  |
| Nocturnal SBP fall, % | 10.84±9.00 | 11.68±7.72 | 0.513 |
| Nocturnal DBP fall, % | 14.90±9.38 | 14.47±9.83 | 0.773 |
| Daytime SBP | 121.99±9.64 | 129.20±9.63 | <0.001 |
| Nighttime SBP | 108.35±10.55 | 113.95±11.63 | 0.007 |
| Daytime DBP | 72.48±6.63 | 78.37±5.62 | <0.001 |
| Nighttime DBP | 61.53±7.95 | 66.99±8.86 | 0.001 |
| Daytime PP | 49.45±6.99 | 50.60±7.83 | 0.256 |
| Nighttime PP | 46.83±7.70 | 46.87±7.40 | 0.996 |
| Daytime ARVs | 18.09±5.44 | 21.24±5.88 | 0.001 |
| Nighttime ARVs | 13.89±6.80 | 13.35±6.29 | 0.728 |
| Daytime ARVd | 14.05±4.88 | 16.95±5.61 | 0.024 |
| Nighttime ARVd | 10.30±6.55 | 10.75±4.88 | 0.356 |
| **Cardiac function characteristic** | |  |  |
| EDV, ml | 111.33±21.65 | 102.48±18.82 | 0.001 |
| ESV, ml | 45.53±9.49 | 40.38±10.57 | 0.001 |
| EF, % | 59.33±4.23 | 60.83±5.99 | 0.047 |
| E/A | 1.87±0.59 | 1.43±0.36 | <0.001 |
| E/e’ | 6.58±1.07 | 5.84±1.22 | 0.001 |
| GLS, % | 20.20±2.02 | 20.94±2.57 | 0.056 |
| GCS, % | 25.65±2.70 | 25.17±2.43 | 0.248 |
| Untwisting rate,゜/s | 77.40±35.10 | 98.11±39.36 | 0.002 |
| Torsion,゜ | 11.56±3.41 | 14.33±4.57 | <0.001 |
| Ees, mmHg/ml | 2.49±1.01 | 2.91±0.87 | 0.001 |
| Ea, mmHg/ml | 1.65±0.35 | 1.82±0.38 | 0.005 |
| VAC | 0.69±0.12 | 0.66±0.18 | 0.079 |

Values are presented as mean ± standard deviation.

HA: high altitude; LA: low altitude; AMS: acute mountain sickness; HR: heart rate; BP: blood pressure; SBP: systolic blood pressure; DBP: diastolic blood pressure; PP: pulse pressure; ARVs: average real variability of SBP; ARVd: average real variability of DBP; GLS: global longitudinal strain; GCS: global circumferential strain; EDV: end-diastolic volume; ESV: end-systolic volume; Ees: end-systolic elastance; Ea: effective arterial elastance; VAC: ventricular-arterial coupling; EF: ejection fraction; E/A: peak early diastolic velocity/late diastolic velocity; E/e’ : peak early diastolic velocity/early diastolic velocity.

P value, total individuals at sea level compared with at high altitude.

**Supplement table 2**. **ICC analysis of intra- and interobserver variations.**

| **Variables** | **Intraobserver variation** | | **P value** | **Interobserver variation** | | **P value** |
| --- | --- | --- | --- | --- | --- | --- |
|  | ICC | 95% CI |  | ICC | 95% CI |  |
| MV E | 0.972 | 0.933-0.989 | <0.001 | 0.951 | 0.881-0.980 | <0.001 |
| MV A | 0.982 | 0.956-0.993 | <0.001 | 0.965 | 0.914-0.986 | <0.001 |
| e’ MVL | 0.967 | 0.918-0.987 | <0.001 | 0.934 | 0.835-0.974 | <0.001 |
| e’ MVS | 0.942 | 0.959-0.977 | <0.001 | 0.941 | 0.858-0.976 | <0.001 |
| GLS, % | 0.868 | 0.702-0.945 | <0.001 | 0.827 | 0.583-0.930 | <0.001 |
| GCS, % | 0.925 | 0.824-0.970 | <0.001 | 0.879 | 0.721-0.950 | <0.001 |
| EDV, ml | 0.993 | 0.976-0.998 | <0.001 | 0.990 | 0.940-0.997 | <0.001 |
| ESV, ml | 0.977 | 0.943-0.991 | <0.001 | 0.953 | 0.878-0.982 | <0.001 |
| Torsion,゜ | 0.935 | 0.845-0.973 | <0.001 | 0.897 | 0.761-0.958 | <0.001 |
| Untwisting rate,゜/s | 0.948 | 0.873-0.979 | <0.001 | 0.910 | 0.779-0.964 | <0.001 |

95% CI, 95% confidence interval;

MV: mitral valve; E: peak early diastolic velocity; A: peak late diastolic velocity; e’: peak early diastolic velocity at the mitral annular; MVL: lateral wall of mitral annulus; MVS: septal wall of mitral annulus; GLS: global longitudinal strain; GCS: global circumferential strain; EDV: end-diastolic volume; ESV: end-systolic volume.
